# 2-Bromo-4-(3,4-dimethyl-5-phenyl-1,3-oxazolidin-2-yl)-6-meth­oxy­phenol

**DOI:** 10.1107/S1600536811051269

**Published:** 2011-12-07

**Authors:** Maywan Hariono, Nurziana Ngah, Habibah A. Wahab, Aisyah Saad Abdul Rahim

**Affiliations:** a School of Pharmaceutical Sciences, Universiti Sains Malaysia, 11800 USM Penang, Malaysia; b Kulliyyah of Science, International Islamic University Malaysia, Bandar Indera Mahkota, 25200 Kuantan, Pahang, Malaysia

## Abstract

In the title compound, C_18_H_20_BrNO_3_, the oxazolidine ring adopts an envelope conformation with the N atom at the flap position. The mean plane of oxazolidine ring makes dihedral angles of 82.96 (13) and 70.97 (12)°, respectively, with the phenyl and benzene rings. In the crystal, adjacent mol­ecules are connected *via* O—H⋯O and C—H⋯O hydrogen bonds and C—H⋯π inter­actions into a zigzag chain along the *b* axis.

## Related literature

For the synthesis and closely related structures, see: Asaruddin *et al.* (2010 ▶); Diwischeck *et al.* (2003 ▶); Khruscheva *et al.* (1997 ▶); Duffy *et al.* (2004 ▶). For therapeutic properties of oxazolidine derivatives, see: Moloney *et al.* (1998 ▶); Wang *et al.* (2010 ▶); Nakano *et al.* (2010 ▶); Fülöp *et al.* (2004 ▶); Panneerselvam (2011 ▶). For standard bond lengths, see: Allen *et al.* (1987 ▶). For the low-temperature device used in the data collection, see: Cosier & Glazer (1986 ▶).
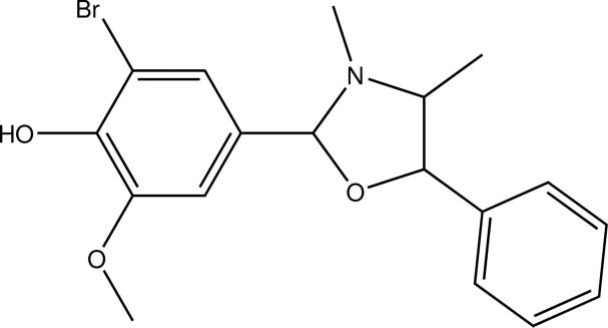


## Experimental

### 

#### Crystal data

C_18_H_20_BrNO_3_
*M* *_r_* = 378.26
Orthorhombic, 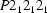

*a* = 7.8056 (4) Å
*b* = 11.9034 (6) Å
*c* = 18.9109 (9) Å
*V* = 1757.07 (15) Å^3^
*Z* = 4
Mo *K*α radiation
μ = 2.35 mm^−1^
*T* = 100 K
0.50 × 0.36 × 0.23 mm

#### Data collection

Bruker SMART APEXII CCD area-detector diffractometer
Absorption correction: multi-scan (*SADABS*; Bruker, 2009 ▶) *T*_min_ = 0.383, *T*_max_ = 0.618
10569 measured reflections
3074 independent reflections
2935 reflections with *I* > 2σ(*I*)
*R*_int_ = 0.037

#### Refinement

*R*[*F* ^2^ > 2σ(*F* ^2^)] = 0.023
*wR*(*F* ^2^) = 0.056
*S* = 1.08
3074 reflections
215 parameters
1 restraint
H atoms treated by a mixture of independent and constrained refinement
Δρ_max_ = 0.32 e Å^−3^
Δρ_min_ = −0.26 e Å^−3^
Absolute structure: Flack (1983 ▶), 1283 Friedel pairs
Flack parameter: 0.004 (7)

### 

Data collection: *APEX2* (Bruker, 2009 ▶); cell refinement: *SAINT* (Bruker, 2009 ▶); data reduction: *SAINT*; program(s) used to solve structure: *SHELXTL* (Sheldrick, 2008 ▶); program(s) used to refine structure: *SHELXTL*; molecular graphics: *SHELXTL*; software used to prepare material for publication: *SHELXTL* and *PLATON* (Spek, 2009 ▶).

## Supplementary Material

Crystal structure: contains datablock(s) global, I. DOI: 10.1107/S1600536811051269/is5016sup1.cif

Structure factors: contains datablock(s) I. DOI: 10.1107/S1600536811051269/is5016Isup2.hkl

Supplementary material file. DOI: 10.1107/S1600536811051269/is5016Isup3.cml

Additional supplementary materials:  crystallographic information; 3D view; checkCIF report

## Figures and Tables

**Table 1:** Hydrogen-bond geometry (Å, °) *Cg*2 is the centroid of the C1–C6 phenyl ring.

*D*—H⋯*A*	*D*—H	H⋯*A*	*D*⋯*A*	*D*—H⋯*A*
O2—H2⋯O1^i^	0.85 (1)	2.03 (1)	2.7853 (19)	148 (2)
C15—H15*A*⋯O2^ii^	0.95	2.46	3.232 (3)	138
C18—H18*A*⋯*Cg*2^i^	0.98	2.96	3.679 (3)	131

Symmetry codes: (i) 
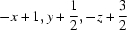
; (ii) 
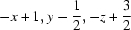
.

**Fig. 1.**
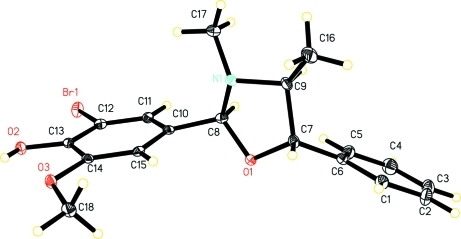
The molecular structure of the title compound, with displacement ellipsoids are drawn at the 50% probability level.

**Fig. 2.**
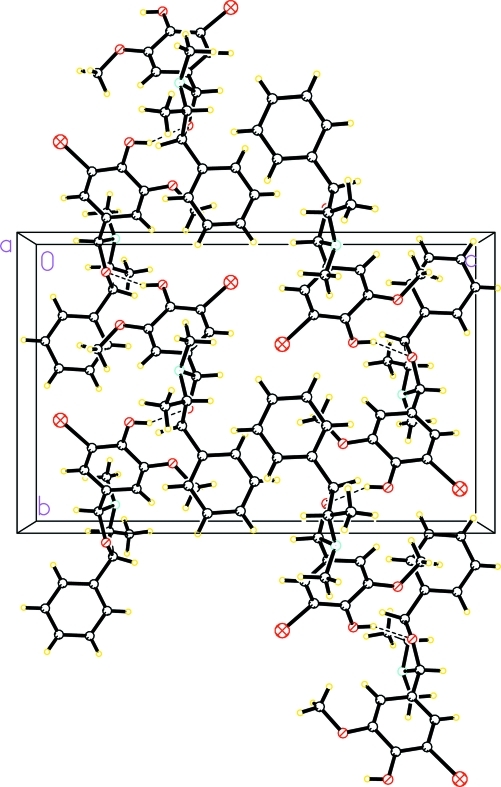
The molecular packing of the title compound viewed down the a axis.

## supplementary crystallographic information

### Comment

Oxazolidine compounds are important in understanding drug behaviour in medicinal chemistry (Duffy *et al.*, 2004). Derivatives of oxazolidine have shown inhibitory effects for several diseases or condition such as β-adrenoreseptor antagonist (Moloney *et al.*, 1998), influenza antiviral (Wang *et al.*, 2010; Nakano *et al.*, 2010), antinflammatory agents (Fulop *et al.*, 2004) and antihyperglycemic (Panneerselvam, 2011). In this paper, we report the X-ray crystal structure of the title oxazolidine compound, (I).

The title compound, C_18_H_20_BrNO_3_, consists of two aromatic rings which are connected through oxazolidine ring (Fig. 1). The molecule is similar with those reported by Asaruddin *et al.* (2010), in that only the present of Br atom at β position of 3-hydroxy-4-methoxyphenyl ring is different. The oxazolidine ring (O1/C7–C9/N1) adopts an envelope conformation with puckering parameters of Q = 0.433 (2) Å and φ = 107.3 (3)°. The N1 atom is at the flap position and it deviates from the mean plane through the remaining four atoms by 0.651 (2) Å. The C1–C6 phenyl and C10–C15 benzene rings make dihedral angles of 82.96 (13) and 70.97 (12)°, respectively, with the mean plane of oxazolidine ring. The bond lengths and angles are in normal ranges (Allen *et al.*, 1987) and in agreement with those reported by Asaruddin *et al.* (2010).

In the crystal structure, adjacent molecules are connected *via* intermolecular O2—H2···O1 and C15—H15A···O2 hydrogen bonds and C18—H18A···*Cg*2 interactions (Table 1) to form a chain along the [010] direction; *Cg*2 is the centroid of the C1–C6 ring.

### Experimental

Following a modified method (Asaruddin *et al.*, 2010; Diwischeck *et al.*, 2003; Khruscheva *et al.*, 1997), (1*S*,2*S*)-2-methylamino-1-phenylpropan-1-ol (0.17 g, 1 mmol) was mixed with 3-bromo-4-hydroxy-5-methoxybenzaldehyde (0.23 g, 1 mmol) in a two-round neck bottom flask. The mixture was dissolved in methanol (4 ml) and molecular sieve 4Å (0.1 g) was added to the reaction mixture then the solution was refluxed at 333 K for 6 h. The solution was filtered and the solvent was evaporated *in vacuo* to give a crude product which was then recrystallized three times from methanol to give colourless blocks with a yield 11%. These were washed with *n*-hexane and dried overnight to afford single crystals suitable for X-ray analysis.

### Refinement

X-ray data were collected at 100 K (Cosier & Glazer, 1986). The hydroxyl H atom was located in a difference map and refined freely [O2—H2 = 0.8499 (10) Å]. Other H atoms were positioned geometrically and refined using riding model with C—H = 0.95–1.00 Å and *U*_iso_(H)=1.2 or 1.5*U*_eq_(C). A rotating group model was applied for methyl group.

### Crystal data

**Table:**

C_18_H_20_BrNO_3_	*F*(000) = 776
*M**_r_* = 378.26	*D*_x_ = 1.430 Mg m^−^^3^
Orthorhombic, *P*2_1_2_1_2_1_	Mo *K*α radiation, λ = 0.71073 Å
Hall symbol: P 2ac 2ab	Cell parameters from 8965 reflections
*a* = 7.8056 (4) Å	θ = 2.0–24.9°
*b* = 11.9034 (6) Å	µ = 2.35 mm^−^^1^
*c* = 18.9109 (9) Å	*T* = 100 K
*V* = 1757.07 (15) Å^3^	Block, colourless
*Z* = 4	0.50 × 0.36 × 0.23 mm

### Data collection

**Table:**

Bruker SMART APEXII CCD area-detector diffractometer	3074 independent reflections
Radiation source: fine-focus sealed tube	2935 reflections with *I* > 2σ(*I*)
graphite	*R*_int_ = 0.037
Detector resolution: 83.66 pixels mm^-1^	θ_max_ = 24.9°, θ_min_ = 2.0°
φ and ω scan	*h* = −9→9
Absorption correction: multi-scan (*SADABS*; Bruker, 2009)	*k* = −14→14
*T*_min_ = 0.383, *T*_max_ = 0.618	*l* = −22→22
10569 measured reflections	

### Refinement

**Table:**

Refinement on *F*^2^	Secondary atom site location: difference Fourier map
Least-squares matrix: full	Hydrogen site location: inferred from neighbouring sites
*R*[*F*^2^ > 2σ(*F*^2^)] = 0.023	H atoms treated by a mixture of independent and constrained refinement
*wR*(*F*^2^) = 0.056	*w* = 1/[σ^2^(*F*_o_^2^) + (0.0197*P*)^2^] where *P* = (*F*_o_^2^ + 2*F*_c_^2^)/3
*S* = 1.08	(Δ/σ)_max_ = 0.001
3074 reflections	Δρ_max_ = 0.32 e Å^−^^3^
215 parameters	Δρ_min_ = −0.26 e Å^−^^3^
1 restraint	Absolute structure: Flack (1983), 1283 Friedel pairs
Primary atom site location: structure-invariant direct methods	Flack parameter: 0.004 (7)

### Special details

**Table:**

Experimental. The crystal was placed in the cold stream of an Oxford Cryosystems Cobra open=flow nitrogen cryostat (Cosier & Glazer, 1986) operating at 100.0 (1) K.
Geometry. All e.s.d.'s (except the e.s.d. in the dihedral angle between two l.s. planes) are estimated using the full covariance matrix. The cell e.s.d.'s are taken into account individually in the estimation of e.s.d.'s in distances, angles and torsion angles; correlations between e.s.d.'s in cell parameters are only used when they are defined by crystal symmetry. An approximate (isotropic) treatment of cell e.s.d.'s is used for estimating e.s.d.'s involving l.s. planes.
Refinement. Refinement of *F*^2^ against ALL reflections. The weighted *R*-factor *wR* and goodness of fit *S* are based on *F*^2^, conventional *R*-factors *R* are based on *F*, with *F* set to zero for negative *F*^2^. The threshold expression of *F*^2^ > σ(*F*^2^) is used only for calculating *R*-factors(gt) *etc*. and is not relevant to the choice of reflections for refinement. *R*-factors based on *F*^2^ are statistically about twice as large as those based on *F*, and *R*- factors based on ALL data will be even larger.

### Fractional atomic coordinates and isotropic or equivalent isotropic displacement parameters (Å^2^)

**Table:**

	*x*	*y*	*z*	*U*_iso_*/*U*_eq_	
Br1	0.58037 (3)	0.865565 (17)	0.942297 (10)	0.02402 (8)	
O1	0.2217 (2)	0.40943 (12)	0.84900 (7)	0.0161 (3)	
O2	0.6507 (2)	0.85865 (13)	0.78478 (7)	0.0187 (3)	
O3	0.5513 (2)	0.71024 (11)	0.68917 (7)	0.0195 (3)	
N1	0.0154 (3)	0.54199 (15)	0.83295 (9)	0.0179 (4)	
C1	0.0051 (3)	0.1430 (2)	0.85221 (12)	0.0259 (5)	
H1A	−0.0140	0.1381	0.8027	0.031*	
C2	−0.0108 (4)	0.0473 (2)	0.89404 (16)	0.0362 (7)	
H2A	−0.0416	−0.0224	0.8732	0.043*	
C3	0.0182 (4)	0.0542 (2)	0.96587 (14)	0.0365 (7)	
H3A	0.0072	−0.0110	0.9944	0.044*	
C4	0.0633 (4)	0.1557 (2)	0.99672 (12)	0.0341 (6)	
H4A	0.0831	0.1600	1.0462	0.041*	
C5	0.0792 (4)	0.25110 (19)	0.95494 (11)	0.0257 (5)	
H5A	0.1110	0.3205	0.9760	0.031*	
C6	0.0490 (3)	0.24580 (18)	0.88251 (10)	0.0191 (5)	
C7	0.0610 (3)	0.34994 (17)	0.83700 (10)	0.0170 (5)	
H7A	0.0563	0.3265	0.7862	0.020*	
C8	0.1793 (3)	0.52295 (17)	0.86771 (10)	0.0165 (5)	
H8A	0.1630	0.5277	0.9201	0.020*	
C9	−0.0781 (3)	0.43775 (18)	0.84969 (10)	0.0191 (5)	
H9A	−0.1098	0.4379	0.9009	0.023*	
C10	0.3140 (3)	0.60567 (17)	0.84574 (10)	0.0152 (5)	
C11	0.3775 (3)	0.68157 (17)	0.89478 (11)	0.0167 (5)	
H11A	0.3434	0.6764	0.9429	0.020*	
C12	0.4899 (3)	0.76443 (18)	0.87389 (10)	0.0162 (5)	
C13	0.5436 (3)	0.77558 (17)	0.80434 (10)	0.0153 (4)	
C14	0.4837 (3)	0.69524 (17)	0.75520 (10)	0.0149 (4)	
C15	0.3686 (3)	0.61278 (17)	0.77486 (10)	0.0153 (4)	
H15A	0.3264	0.5611	0.7407	0.018*	
C16	−0.2384 (3)	0.4204 (2)	0.80546 (13)	0.0302 (6)	
H16A	−0.3266	0.4741	0.8202	0.045*	
H16B	−0.2808	0.3437	0.8122	0.045*	
H16C	−0.2112	0.4324	0.7554	0.045*	
C17	−0.0712 (3)	0.64373 (18)	0.85614 (11)	0.0258 (5)	
H17A	0.0013	0.7090	0.8461	0.039*	
H17B	−0.0931	0.6394	0.9071	0.039*	
H17C	−0.1802	0.6513	0.8309	0.039*	
C18	0.5220 (3)	0.62188 (19)	0.63849 (10)	0.0224 (5)	
H18A	0.5904	0.6361	0.5960	0.034*	
H18B	0.4003	0.6199	0.6259	0.034*	
H18C	0.5554	0.5496	0.6591	0.034*	
H2	0.659 (3)	0.856 (2)	0.74000 (15)	0.022 (6)*	

### Atomic displacement parameters (Å^2^)

**Table:**

	*U*^11^	*U*^22^	*U*^33^	*U*^12^	*U*^13^	*U*^23^
Br1	0.03139 (14)	0.02299 (12)	0.01767 (10)	−0.00772 (11)	−0.00524 (10)	−0.00103 (8)
O1	0.0127 (8)	0.0156 (7)	0.0200 (7)	−0.0001 (7)	0.0011 (6)	0.0004 (5)
O2	0.0203 (8)	0.0183 (8)	0.0176 (7)	−0.0032 (7)	0.0025 (6)	0.0005 (6)
O3	0.0228 (10)	0.0188 (8)	0.0171 (6)	−0.0038 (7)	0.0049 (7)	−0.0016 (5)
N1	0.0126 (10)	0.0177 (10)	0.0234 (8)	−0.0018 (8)	−0.0014 (8)	0.0019 (7)
C1	0.0216 (13)	0.0234 (12)	0.0328 (11)	−0.0021 (11)	−0.0001 (10)	−0.0009 (10)
C2	0.0318 (16)	0.0194 (13)	0.0574 (16)	−0.0070 (12)	0.0011 (14)	0.0022 (11)
C3	0.0310 (16)	0.0269 (14)	0.0517 (15)	−0.0027 (12)	0.0041 (13)	0.0184 (11)
C4	0.0358 (16)	0.0388 (15)	0.0278 (11)	0.0018 (14)	0.0042 (12)	0.0111 (10)
C5	0.0295 (14)	0.0222 (11)	0.0252 (10)	0.0001 (11)	0.0019 (12)	0.0020 (8)
C6	0.0132 (13)	0.0196 (11)	0.0245 (10)	0.0004 (10)	0.0034 (10)	0.0023 (8)
C7	0.0159 (12)	0.0190 (11)	0.0160 (8)	−0.0046 (10)	−0.0001 (9)	−0.0016 (8)
C8	0.0167 (13)	0.0180 (11)	0.0150 (9)	0.0035 (10)	−0.0007 (9)	−0.0005 (8)
C9	0.0143 (11)	0.0219 (11)	0.0211 (9)	−0.0030 (11)	0.0023 (10)	0.0026 (8)
C10	0.0110 (11)	0.0163 (11)	0.0184 (9)	0.0043 (9)	−0.0003 (8)	0.0024 (8)
C11	0.0165 (12)	0.0177 (10)	0.0159 (9)	0.0022 (9)	−0.0006 (9)	0.0031 (8)
C12	0.0146 (12)	0.0173 (11)	0.0167 (9)	0.0016 (9)	−0.0039 (9)	−0.0036 (8)
C13	0.0105 (12)	0.0149 (10)	0.0204 (9)	0.0012 (9)	−0.0012 (9)	0.0026 (7)
C14	0.0118 (11)	0.0167 (11)	0.0163 (9)	0.0040 (9)	0.0005 (9)	0.0015 (8)
C15	0.0149 (11)	0.0136 (11)	0.0174 (9)	0.0034 (9)	−0.0026 (9)	−0.0004 (7)
C16	0.0171 (13)	0.0353 (14)	0.0383 (12)	−0.0027 (12)	−0.0068 (12)	0.0025 (10)
C17	0.0178 (12)	0.0250 (12)	0.0345 (11)	0.0039 (14)	0.0010 (11)	0.0037 (9)
C18	0.0256 (12)	0.0229 (12)	0.0186 (9)	−0.0033 (11)	0.0040 (9)	−0.0058 (9)

### Geometric parameters (Å, °)

**Table:**

Br1—C12	1.903 (2)	C7—H7A	1.0000
O1—C8	1.435 (3)	C8—C10	1.499 (3)
O1—C7	1.458 (3)	C8—H8A	1.0000
O2—C13	1.347 (3)	C9—C16	1.519 (4)
O2—H2	0.8499 (10)	C9—H9A	1.0000
O3—C14	1.367 (2)	C10—C11	1.386 (3)
O3—C18	1.441 (2)	C10—C15	1.409 (3)
N1—C17	1.455 (3)	C11—C12	1.378 (3)
N1—C8	1.456 (3)	C11—H11A	0.9500
N1—C9	1.474 (3)	C12—C13	1.387 (3)
C1—C2	1.392 (3)	C13—C14	1.413 (3)
C1—C6	1.394 (3)	C14—C15	1.382 (3)
C1—H1A	0.9500	C15—H15A	0.9500
C2—C3	1.379 (4)	C16—H16A	0.9800
C2—H2A	0.9500	C16—H16B	0.9800
C3—C4	1.387 (4)	C16—H16C	0.9800
C3—H3A	0.9500	C17—H17A	0.9800
C4—C5	1.389 (3)	C17—H17B	0.9800
C4—H4A	0.9500	C17—H17C	0.9800
C5—C6	1.391 (3)	C18—H18A	0.9800
C5—H5A	0.9500	C18—H18B	0.9800
C6—C7	1.512 (3)	C18—H18C	0.9800
C7—C9	1.526 (3)		
			
C8—O1—C7	107.31 (16)	N1—C9—H9A	109.2
C13—O2—H2	107.2 (18)	C16—C9—H9A	109.2
C14—O3—C18	116.80 (16)	C7—C9—H9A	109.2
C17—N1—C8	113.71 (18)	C11—C10—C15	119.30 (19)
C17—N1—C9	113.94 (18)	C11—C10—C8	119.56 (18)
C8—N1—C9	101.95 (17)	C15—C10—C8	120.99 (18)
C2—C1—C6	120.4 (2)	C12—C11—C10	120.15 (19)
C2—C1—H1A	119.8	C12—C11—H11A	119.9
C6—C1—H1A	119.8	C10—C11—H11A	119.9
C3—C2—C1	119.8 (2)	C11—C12—C13	122.20 (19)
C3—C2—H2A	120.1	C11—C12—Br1	119.62 (15)
C1—C2—H2A	120.1	C13—C12—Br1	118.16 (16)
C2—C3—C4	120.5 (2)	O2—C13—C12	121.22 (18)
C2—C3—H3A	119.7	O2—C13—C14	121.44 (18)
C4—C3—H3A	119.7	C12—C13—C14	117.32 (19)
C3—C4—C5	119.7 (2)	O3—C14—C15	126.11 (18)
C3—C4—H4A	120.2	O3—C14—C13	112.63 (18)
C5—C4—H4A	120.2	C15—C14—C13	121.26 (18)
C4—C5—C6	120.5 (2)	C14—C15—C10	119.68 (19)
C4—C5—H5A	119.7	C14—C15—H15A	120.2
C6—C5—H5A	119.7	C10—C15—H15A	120.2
C5—C6—C1	119.1 (2)	C9—C16—H16A	109.5
C5—C6—C7	120.85 (18)	C9—C16—H16B	109.5
C1—C6—C7	120.07 (18)	H16A—C16—H16B	109.5
O1—C7—C6	111.27 (17)	C9—C16—H16C	109.5
O1—C7—C9	104.75 (16)	H16A—C16—H16C	109.5
C6—C7—C9	115.34 (19)	H16B—C16—H16C	109.5
O1—C7—H7A	108.4	N1—C17—H17A	109.5
C6—C7—H7A	108.4	N1—C17—H17B	109.5
C9—C7—H7A	108.4	H17A—C17—H17B	109.5
O1—C8—N1	103.74 (17)	N1—C17—H17C	109.5
O1—C8—C10	112.86 (18)	H17A—C17—H17C	109.5
N1—C8—C10	112.88 (17)	H17B—C17—H17C	109.5
O1—C8—H8A	109.1	O3—C18—H18A	109.5
N1—C8—H8A	109.1	O3—C18—H18B	109.5
C10—C8—H8A	109.1	H18A—C18—H18B	109.5
N1—C9—C16	113.81 (18)	O3—C18—H18C	109.5
N1—C9—C7	101.0 (2)	H18A—C18—H18C	109.5
C16—C9—C7	113.98 (18)	H18B—C18—H18C	109.5
			
C6—C1—C2—C3	−0.5 (5)	C6—C7—C9—N1	149.13 (17)
C1—C2—C3—C4	0.0 (5)	O1—C7—C9—C16	148.90 (18)
C2—C3—C4—C5	0.0 (5)	C6—C7—C9—C16	−88.4 (2)
C3—C4—C5—C6	0.5 (5)	O1—C8—C10—C11	−129.0 (2)
C4—C5—C6—C1	−1.0 (4)	N1—C8—C10—C11	113.7 (2)
C4—C5—C6—C7	178.2 (3)	O1—C8—C10—C15	55.5 (3)
C2—C1—C6—C5	1.0 (4)	N1—C8—C10—C15	−61.8 (3)
C2—C1—C6—C7	−178.2 (2)	C15—C10—C11—C12	1.4 (3)
C8—O1—C7—C6	−124.92 (17)	C8—C10—C11—C12	−174.2 (2)
C8—O1—C7—C9	0.36 (18)	C10—C11—C12—C13	0.0 (3)
C5—C6—C7—O1	50.7 (3)	C10—C11—C12—Br1	−178.39 (17)
C1—C6—C7—O1	−130.1 (2)	C11—C12—C13—O2	178.6 (2)
C5—C6—C7—C9	−68.4 (3)	Br1—C12—C13—O2	−3.0 (3)
C1—C6—C7—C9	110.7 (2)	C11—C12—C13—C14	−2.5 (3)
C7—O1—C8—N1	−27.71 (18)	Br1—C12—C13—C14	175.97 (16)
C7—O1—C8—C10	−150.23 (16)	C18—O3—C14—C15	−10.8 (3)
C17—N1—C8—O1	167.85 (16)	C18—O3—C14—C13	169.59 (19)
C9—N1—C8—O1	44.77 (18)	O2—C13—C14—O3	2.2 (3)
C17—N1—C8—C10	−69.6 (2)	C12—C13—C14—O3	−176.71 (19)
C9—N1—C8—C10	167.27 (17)	O2—C13—C14—C15	−177.4 (2)
C17—N1—C9—C16	71.3 (2)	C12—C13—C14—C15	3.6 (3)
C8—N1—C9—C16	−165.77 (19)	O3—C14—C15—C10	178.0 (2)
C17—N1—C9—C7	−166.13 (17)	C13—C14—C15—C10	−2.3 (3)
C8—N1—C9—C7	−43.20 (18)	C11—C10—C15—C14	−0.2 (3)
O1—C7—C9—N1	26.45 (18)	C8—C10—C15—C14	175.3 (2)

### Hydrogen-bond geometry (Å, °)

**Table:**

*Cg*2 is the centroid of the C1–C6 phenyl ring.

**Table:**

*D*—H···*A*	*D*—H	H···*A*	*D*···*A*	*D*—H···*A*
O2—H2···O1^i^	0.85 (1)	2.03 (1)	2.7853 (19)	148 (2)
C15—H15A···O2^ii^	0.95	2.46	3.232 (3)	138
C18—H18A···Cg2^i^	0.98	2.96	3.679 (3)	131

Symmetry codes: (i) −*x*+1, *y*+1/2, −*z*+3/2; (ii) −*x*+1, *y*−1/2, −*z*+3/2.
